# The Fabrication and Indentation of Cubic Silicon Carbide Diaphragm for Acoustic Sensing

**DOI:** 10.3390/mi12091101

**Published:** 2021-09-13

**Authors:** Siti Aisyah Zawawi, Azrul Azlan Hamzah, Burhanuddin Yeop Majlis, Faisal Mohd-Yasin

**Affiliations:** 1 Institute of Microengineering and Nanoelectronics, Universiti Kebangsaan Malaysia, Bangi 43600, Selangor, Malaysia; aisyahzawawi@gmail.com (S.A.Z.); azlanhamzah@ukm.edu.my (A.A.H.); burhan@ukm.edu.my (B.Y.M.); 2 UiTM Foundation Centre, Dengkil Campus, Universiti Teknologi Mara, Dengkil 43800, Selangor, Malaysia; 3 Queensland Micro- and Nanotechnology Centre, Griffith University, Nathan, QLD 4111, Australia

**Keywords:** indentation, silicon carbide, diaphragm, load-displacement curve, microphone

## Abstract

In this study, 550 nm thick cubic silicon carbide square diaphragms were back etched from Si substrate. Then, indentation was carried out to samples with varying dimensions, indentation locations, and loads. The influence of three parameters is documented by analyzing load-displacement curves. It was found that diaphragms with bigger area, indented at the edge, and low load demonstrated almost elastic behaviour. Furthermore, two samples burst and one of them displayed pop-in behaviour, which we determine is due to plastic deformation. Based on optimum dimension and load, we calculate maximum pressure for elastic diaphragms. This pressure is sufficient for cubic silicon carbide diaphragms to be used as acoustic sensors to detect poisonous gasses.

## 1. Introduction

Silicon carbide (SiC) has great potential as a material for micro-electromechanical systems (MEMS) due to its excellent mechanical properties such as high hardness, excellent wear resistance [1], high Young’s Modulus [2,3], and operation at high temperatures of up to 500 °C [4]. This material is available in bulk or as thin film on top of the silicon (Si) substrate. For the latter case, it is cheaper in cost but would need a protective package to protect the Si die for operation in extreme environments. For any material, if one would like to employ its freestanding thin film as a sensor, one should take into consideration that the mechanical properties are different from bulk material. This is due to the size effect that occurs from the reduction of film thickness [5,6], and the surface effect due to the microstructural changes [7]. Furthermore, multilayer or composite thin film has different residual stress in comparison to single layer film [8]. Therefore, specific tests should be conducted to find the mechanical properties of freestanding thin film instead of using data from bulk material.

Different methods have been employed to investigate the mechanical properties of freestanding thin films. These include bending test [9], fracture test [10], micro-tensile test [11], bulge test [12], and combined bending and strain tests [13]. Some of these works employ indentation equipment to deflect the freestanding thin film. This equipment supplies a precise amount of force to indent the film and record its vertical displacement. Then, the load-displacement graph is produced by the software [14]. The mechanical response of freestanding thin film of various materials was studied using indentation, namely Au [11], Al_2_O_3_ [12], SiN [15], polysilicon [16], and Al [17]. Based on our literature search, Pecholt and Molian [18] was the only study that used indentation to characterize the mechanical properties of cubic silicon carbide (3C-SiC) freestanding thin films. Specifically, they compared the values of Young’s modulus and fracture strength of SiC cantilevers that were fabricated by laser etching and reactive ion etching. Besides indentation, the mechanical properties of SiC thin films have been measured by other methods. Its flexural strength was determined by a three point bending test [19,20,21], and its hardness by Vicker’s hardness tester and uniaxial tensile test [19,20]. Hayun et al. [19] determined the elastic modulus of SiC bulk by using the data from the ultrasonic velocity and the density that were measured using the liquid displacement method. Gubernat et al. [21] investigated the Young’s Modulus, Kirchoff modulus and Poisson ratio of SiC powder using the ultrasonic method. Cheng et al. [2] found the mechanical properties of SiC nanowires using a tensile test.

Beside the freestanding 3C-SiC cantilever, the use of a 3C-SiC diaphragm in MEMS sensors is equally important, especially for applications in extreme environments [1,4]. In terms of its use as an acoustic sensor, we performed a comprehensive literature review on the articles that published prototypes of MEMS microphones in the past 30 years [22]. We found that silicon carbide has never been employed as the acoustic diaphragm. Owing to its superior mechanical and chemical properties, a silicon carbide-based microphone could be used in extreme environments, for example, in detecting toxic gasses at elevated temperatures. That is the main novelty of our project. In our 2019 article, we started by utilizing the Micro Materials Nanotest® indentation system to determine the hardness, tensile modulus, shear strength, shear modulus and tensile stress of the 3C-SiC film on top of the Si substrate [23].

In this latest work, we back-etched the Si substrate to create the freestanding 3C-SiC diaphragms. The fabrication process and image of the samples are provided in Section 2. As the main objective is to determine whether the diaphragms are suitable for application as an acoustic sensor, Section 3 describes the indentation that was carried out to six samples with different dimensions, indentation location, and maximum load. The results of the indentation are given in Section 4. We plot the load-displacement curves for all samples and provide our observations on the influence of the diaphragm’s dimension, indentation location, and maximum load. Two samples were damaged, so we analytically investigate the cause in Section 4.3. After that, we return to the main objective of this work in Section 4.5, which is to determine the maximum pressure that the 3C-SiC diaphragms could endure for the intended application. Section 5 concludes this paper.

## 2. Fabrication of Freestanding Diaphragms

**Figure 1:**
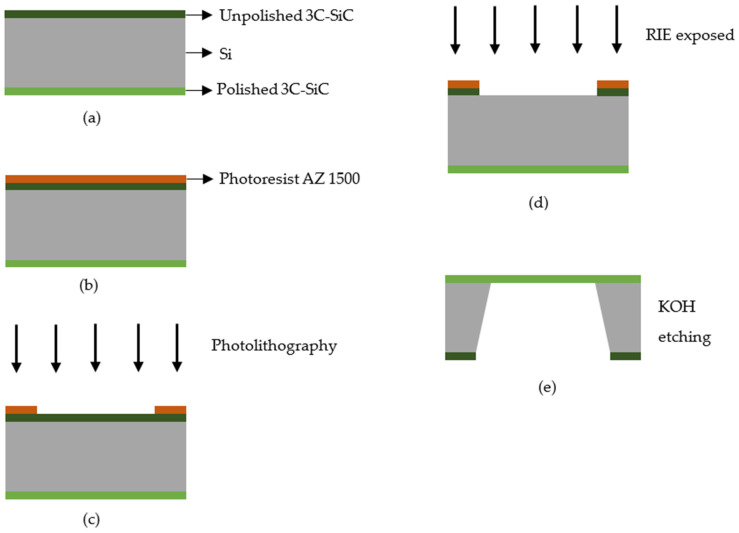
The fabrication process for the back-etching of cubic silicon carbide diaphragm from silicon substrate is shown from step (**a**–**e**).

The 3C-SiC-on-Si substrate was previously deposited by Wang et al. [24,25,26] at Queensland Micro and Nanotechnology Centre of Griffith University. The characterization data from those articles demonstrate that these groups obtained a single crystal 3C-SiC film with no apparent defect. It should be noted that the authors of this paper are not involved with that deposition. Nevertheless, the fabrication summary is provided herein. The epitaxial 3C-SiC film was grown on top of Si substrate by low pressure chemical vapor deposition (LPCVD) using precursors gasses silane (SiH_4_) and propylene (C_3_H_6_). The deposition temperature was fixed at 1000 °C. A 3C-SiC layer was deposited on both sides of (100) Si substrate, i.e., polished and unpolished sides as shown in Figure 1a. The thicknesses of 3C-SiC film and Si substrate are 550 nm and 675 mm, respectively.

**Figure 2:**
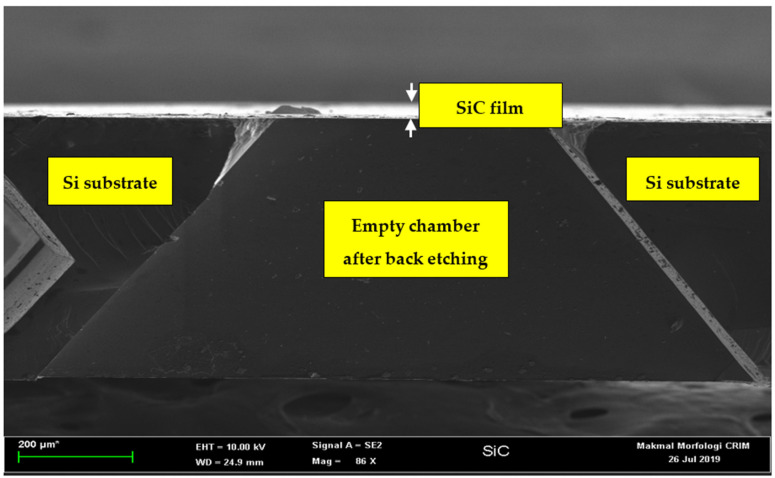
The cross-sectional image of back-etched 3C-SiC diaphragm from Si substrate is shown. The labels help readers to understand the location of the suspended film, the chamber, and the Si substrate.

**Figure 3:**
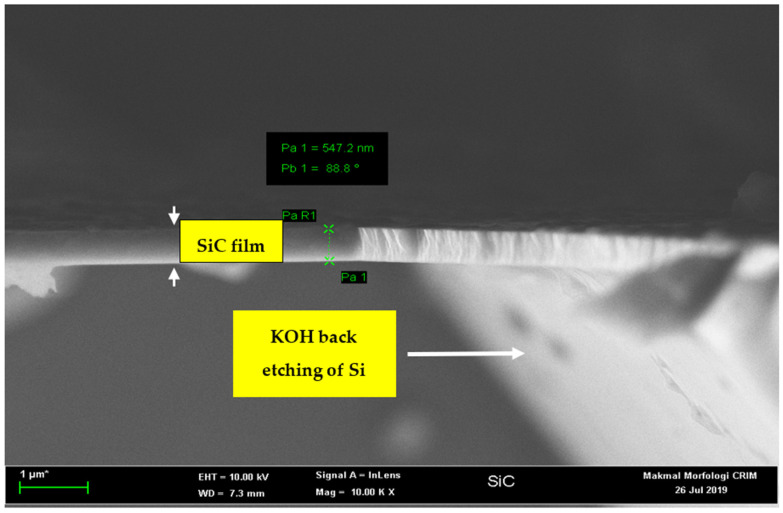
The closed-up view of the suspended 3C-SiC diaphragm is shown. The parameter Pa represents the thickness of the 3C-SiC layer, while Pb is the tilt angle.

**Figure 4:**
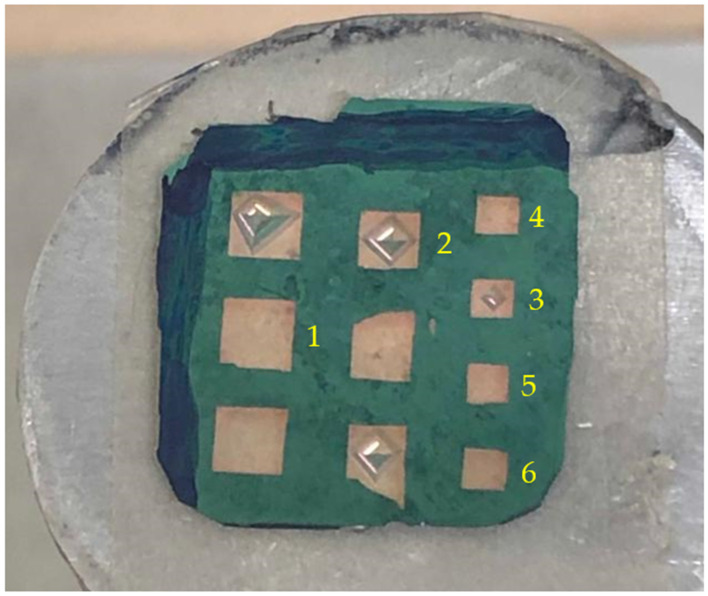
The top view images of all the fabricated samples are shown. Each column represents samples with the same dimensions. Out of 9, only 6 samples were used in the indentation experiment. They are labeled as sample 1 to sample 6.

We have designated the polished side of the 3C-SiC layer as the acoustic diaphragm. Therefore, some part of the unpolished 3C-SiC layer and the Si substrate needs to be removed to pattern the square diaphragms of different dimensions. Figure 1b shows the first step for the deposition of the photoresist. AZ 1500 positive photoresist layer was deposited by using the spin coating machine. The thickness of this photoresist was 1.5 µm. It was spin coated on top of the sample at the rotation speed of 1500 rpm for 10 s, followed by the second spinning at 4000 rpm for 40 s. Then, the sample was baked twice to harden the photoresist. The first baking was at a temperature of 110 °C for 60 s, and the second baking was at a temperature of 220 °C for 60 s. Figure 1c shows the second step to remove the photoresist layer. The photolithography mask is made of glass. The sample was aligned by mask aligner MDA-400LJ (Midas System Co. Ltd, Daejeon, Republic of Korea). It was then exposed to UV light for 35 s to soften the area of the photoresist under the mask. The exposed sample was then immersed in the AZ 300K developer (Integrated Micro Materials, Argyle, TX, USA) for 30 s to remove the softened photoresist. Figure 1d shows the third step to remove the unpolished 3C-SiC layer using the reactive ion etching (RIE) process. Sulfur hexafluoride (SF_6_) and oxygen (O_2_) were used as the etching gasses at the flow rate of 60 sccm and 10 sccm, respectively. The optimum etching time, pressure and RF power to remove the 550 nm thick 3C-SiC film were 45 min, 0.08 Torr and 100 W, respectively. Figure 1e shows the final step to remove the Si substrate. The back-etching of the Si substrate from the 3C-SiC-on-Si sample was carried out by immersing it into the etchant at the concentration of KOH (45%wt) + DI (55%wt) with 10% of IPA. IPA solution was added to assure a smooth 3C-SiC surface [27]. The etching time was approximately 15 h at a temperature of 80 °C. After fabrication, we took the images of these samples. Figure 2 shows the cross-sectional image of the back-etched sample, and Figure 3 is the close-up view of the freestanding diaphragm. From that image, we have independently measured its thickness to be 547 nm. Finally, Figure 4 shows the top view image of square diaphragms. Each column represents samples with the same dimensions. Out of nine fabricated samples, the best six were chosen for the indentation experiment.

## 3. Nanoindentation of Diaphragms

**Figure 5:**
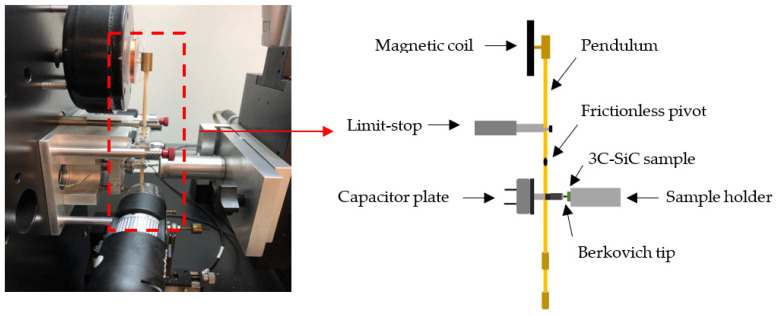
The schematic of overall experimental setup from our 2019 JJAP article [23]. © IOP Publishing. Reproduced with permission. All rights reserved.

The indentation of the diaphragms was performed using the MicroMaterials NanoTest system with a Berkovich three-sided pyramidal diamond tip, as shown in Figure 5. The detailed operation has been described in [23], and is summarized herein. This system employed the ceramic pendulum that was balanced by the frictionless pivot. The limit-stop set the maximum displacement of the diamond tip, by adjusting its position using micrometer. The tip’s radius was 300 nm. The limit-stop also determined the orientation of the pendulum when the load was applied. The system supplied the current through the magnetic coil on top of the pendulum to move the diamond tip towards the sample. The latter was held in place by a sample holder. The displacement of the diamond tip was measured by a capacitive sensor. The sensor is located just below the pivot and comprises of two parallel circular discs. The reading resolution is automatically determined and executed by the system. A computer program recorded all important parameters, namely the maximum load, loading rate, and amount of displacement.

Six fabricated samples were indented, and their identification numbers are denoted in Figure 4. In this paper, the indentation of those samples was conducted under the load control mode. Under this mode, the maximum load was set at three values (10 mN, 3 mN, and 1.5 mN) at the constant loading rate of 0.5 mN/s. In parallel, we also set the diamond tip’s maximum displacement at 20 cm. In operational terms, this indentation system would commence the loading cycle until it reached either the maximum load or maximum distance. Once that happened, it automatically commenced the unloading cycle. The results of the load-displacement curve for both cycles were automatically recorded and plotted, as discussed in Section 4.

## 4. Results and Analysis

The characteristics of the samples and their data from the load-displacement curves are summarized in Table 1. Samples 1 and 2 have different side lengths to investigate the influence of the size of the diaphragm, as detailed in Section 4.1. Section 4.2 describes the result of the indentation test at two different locations. Hence, samples 3 and 4 have the same dimensions, but were indented at the center and edge of their diaphragms, respectively. For sample 4, the indentation location is defined as 30% of the distance from the center to the edge of the sample. Samples 2 and 3 burst, so Section 4.3 investigate the cause. Section 4.4 documents the indentation result for samples 5 and 6 having the same dimension, indentation location, and maximum load. Based on the indentation data, Section 4.5 discusses whether the back-etched 3C-SiC diaphragm would be suitable for the intended application as an acoustic sensor to detect poisonous gasses.

### 4.1. Influence of the Size of the Diaphragm

**Figure 6:**
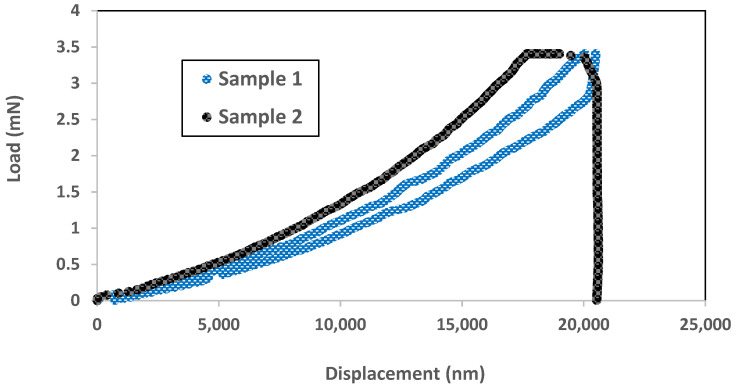
Shows the load-displacement curve for sample1 (blue line) and sample 2 (black line). The side length of the former and latter are 2300 and 1800 µm, respectively. In other words, sample 1 is 63% bigger than the sample 2. The maximum load was set at 10 mN for this experiment.

Figure 6 illustrates the differences in the behavior between the samples of different sizes. First, a bigger diaphragm was more elastic than a smaller diaphragm. This was demonstrated by the completed loading and unloading cycles for sample 1 after reaching the maximum tip displacement of 20 µm, whereas, sample 2 could not complete the unloading cycle as the tip pierces the diaphragm at the load of 3.5 mN. Second, the smaller diaphragm needed a bigger load to achieve the same amount of displacement as the bigger diaphragm. This was due to higher stiffness, as the area of sample 2 is smaller. The third observation is the most important. The bigger diaphragm could sustain a higher load and larger displacement, at the price of more expensive real estate. From the literature search, Espinosa et al. [11] reported the same observation for Au films when they performed indentation with samples of different sizes.

### 4.2. Influence of the Location of Indentation

**Figure 7:**
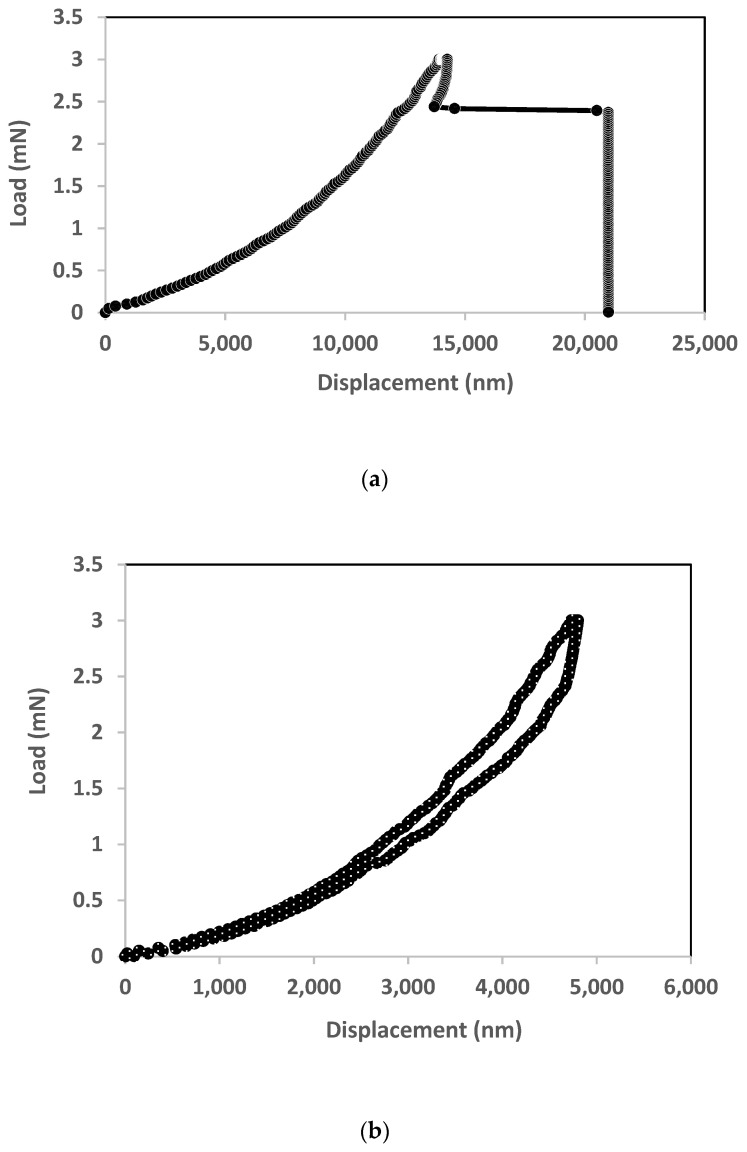
The load-displacement curves for two samples with identical properties and sizes, but were indented at different locations: (**a**) sample 3 was indented at the centre of diaphragm, (**b**) sample 4 was indented at the edge of diaphragm. The maximum load being applied to both samples is 3 mN.

Figure 7 shows the load-displacement curve for samples that are identical in terms of the area (1300 µm × 1300 µm), but were indented at two different locations. The maximum load was set at 3 mN. Figure 7a shows the curve for the diaphragm of sample 3 that was indented at the center, while Figure 7b shows the curve for the diaphragm of sample 4 that was indented at its edge. We can state three observations from both figures. First, sample 4 completed the loading and unloading cycles after reaching a maximum load of 3 mN, whereas sample 3 has burst diaphragm during the unloading cycle. Second, sample 3 has higher displacement (14 µm) than sample 3 (5 µm), albeit being subjected to the same amount of load. The third observation is the most interesting. As can be seen from Figure 7a, pop-in behavior occurred to sample 3, i.e., the indenter suddenly experienced an increase in penetration depth without any major increase in the applied indentation load. According to Vachhani et al. [28], this could happen at any possible point during the loading or unloading cycle. For sample 3, it happened in the latter case, i.e., once the load was reduced to 2.4 mN from the max load of 3 mN. The pop-ins occurred at the displacement of 14.5 µm, drastically shifted it to a maximum displacement of 20 µm. The diaphragm burst afterwards.

Zhao et al. [29] investigated fractured poly SiC film under contact load and reveals that it could be due to the material’s defect. This defect could be due to two factors, i.e., micro-crack and dislocations. The former occurs by “cleavage”, when maximum tensile stress around the indenter tip exceeds theoretical cleavage strength. The latter is induced by onset plastic deformation [30,31]. The next sub-section attempted to determine which factor causes the fracture for sample 3.

### 4.3. Determining the Cause of Burst Diaphragm

Wang et al. [24] state that dislocation as shown in Figure 7a is induced by onset plastic deformation, i.e., maximum shear stress beneath the indenter tip exceeds the theoretical shear strength of 3C-SiC. It is also well known that shear strength is the maximum shear stress developed at the time of failure, in our case when the diaphragm burst. The maximum shear stress ($\tau_{max}$) under the indenter tip can be calculated using the following equation [29]:

(1) τmax=0.31 (6P(Er)2π3R2)13 

where P and R are the maximum load applied (3 mN) and radius of the indenter tip (300 nm for Berkovich tip), respectively. The reduced modulus ( $E_{r}$) was determined from indenter analysis (0.7 GPa). From Equation (1), the value of the shear stress for our 3C-SiC diaphragm under the indenter tip in Figure 7a to be 455 MPa.

The theoretical shear stress can be estimated by Equation (2) [29]:

(2) $$\tau_{th}=\frac{G_{s}}{2\pi }\;$$

where $G_{s}$ is a shear modulus of the material, given by Equation (3) [29]:

(3) $$G_{s}=\frac{E_{s}}{2\;\left(1+v_{s}\right)}\;$$

where $v_{s}$ is a Poisson ratio of the sample and $E_{s}$ is given by Equation (4) [29]:

(4) $$\frac{1}{E_{r}}=\frac{1-{\left(v_{s}\right)}^{2}}{E_{s}}+\;\frac{1-{\left(v_{i}\right)}^{2}}{E_{i}}\;$$

where $E_{s}$ is Young’s Modulus of the 3C-SiC, and $v_{i}$ and $E_{i}$. are Poisson’s ratio and Young’s Modulus of the indenter ($v_{i}$ = 0.07 and $E_{i}$ = 1141 GPa), respectively. Substituting Equations (3) and (4), the theoretical shear stress of the 3C-SiC diaphragm in Figure 7a was 44 MPa. As the diaphragm failed at this time, this parameter is equal to the theoretical shear strength of 3C-SiC. This value was significantly lower than the $\tau_{max}$ under the indenter tip, i.e., 455 MPa. We could therefore deduce that the load/unload curve in Figure 7a underwent plastic deformation.

### 4.4. Load-Displacement Curve for Elastic Diaphragm

**Figure 8:**
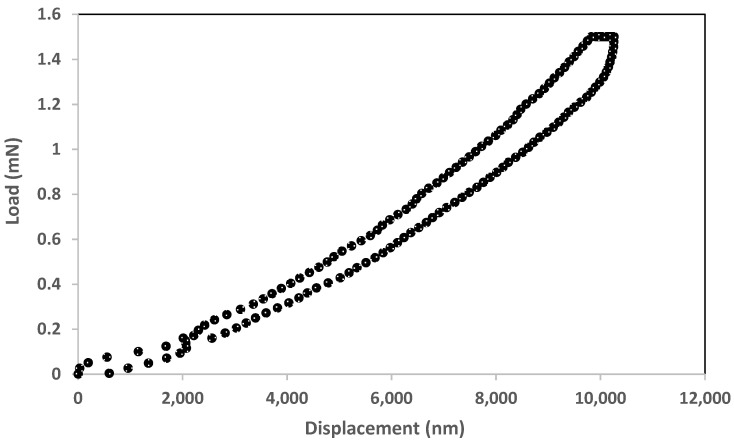
Load-displacement curves for average values of samples 5 and 6. Both are identical in properties and indented at the center. The maximum load is 1.5 mN.

One of the possible applications for the 3C-SiC diaphragm is as an acoustic membrane in a MEMS microphone. For that usage, the sound waves primarily deflect the center of the diaphragm. We have learnt from Section 4.1 and Section 4.2 that the maximum load must be limited to 3 mN to avoid the diaphragms that were indented in the center from being damaged. Figure 8 shows the load-displacement curves that are plotted from the average data points of samples 5 and 6. Both diaphragms are identical in terms of materials (back-etched 3C-SiC diaphragms from the same Si wafer), thickness (550 nm) and area (1300 µm × 1300 µm), and were indented at the center. The maximum load is set to 1.5 mN in this experiment. We observe that the loading and unloading cycles for samples 5 and 6 followed a similar pattern and load/displacement rate, attesting to identical properties, size and indentation location. In order to determine whether the samples have elastic or plastic deformation, we measure the difference in the displacement of the diaphragms at a load of 0 N during the loading and unloading cycles. The value is 0.5 µm. Considering the maximum displacement of 10 µm, this is a difference of 5%. In other words, we could state the samples 5 and 6 have “almost” elastic deformation, which makes them suitable for the intended application.

### 4.5. Suitability of Elastic Diaphragm as Acoustic Sensor

The ultimate goal of this project is to use the SiC diaphragm in a MEMS microphone to detect poisonous gasses [32,33,34]. The data from the indentation experiment show that the maximum load that the diaphragm could take before bursting is 3 mN. Since the area of the diaphragm is 1300 µm, the equivalent pressure is 1.75 kPa. Therefore, we searched the literature to find out the minimum pressure that is required to detect leaked gasses. It was found that two groups, i.e., Ravula et al. [35] and Huang et al. [36], successfully detect gasses at that range. These data convince us of the possible use of our 3C-SiC diaphragms for detecting poisonous gasses. We should also take into consideration the careful design of its signal processing circuitries to accomplish this objective, as they should be low noise [37] and low power [38].

## 5. Conclusions

We performed the fabrication and indentation of freestanding 3C-SiC diaphragms to determine their suitability as acoustic sensors. The samples were back-etched from the custom-made 3C-SiC-on-Si wafer. We indented diaphragms at different dimensions, indentation locations, and maximum loads. The data were plotted as load-displacement curves. It was found that diaphragms with bigger areas, indented at the edge, and low load demonstrated almost elastic behaviour. Furthermore, two samples burst; one of them displayed “pop-in” behaviour, which we analytically determined was due to plastic deformation. Based on the optimum dimension and load that were applied to the elastic diaphragms, we calculated the maximum pressure that they could sustain. A literature review of similar works indicated that the diaphragm of this pressure would be sufficient to be used as an acoustic sensor to detect poisonous gasses.

There are two limitations from this work which warrant further studies. First, since the maximum displacement is 20 µm and the diaphragm’s thickness is only 550 nm, we should have taken into consideration the effect on non-linearity of the displacement of the thin film. Second, the number of samples per experiment should also be increased from one to five, as used by Espinosa et al. [11] to increase the reliability and repeatability of the results.

## Figures and Tables

**Table 1:** Summary of samples under test.

Sample Number	Side Length (µm)	Indent Location	Max Load (mN)	Displacement * (µm)
1	2300	Center	10	20.048
2	1800	Center	10	17.673
3	1300	Center	3	20.979
4	1300	Edge	3	4.801
5	1300	Center	1.5	9.474
6	1300	Center	1.5	11.045

* The maximum displacement for the diamond tip is set at 20 µm.

## Data Availability

The data that support the findings of this study are available from the authors upon reasonable request.
